# How do city-specific factors affect migrant integration in China? A study based on a hierarchical linear model of migrants and cities

**DOI:** 10.1371/journal.pone.0244665

**Published:** 2021-01-12

**Authors:** Rumin Zheng, Lin Mei, Yanhua Guo, Shuo Zhen, Zhanhui Fu

**Affiliations:** 1 Key Laboratory of Geographical Processes and Ecological Security of Changbai Mountains, Ministry of Education, School of Geographical Science, Northeast Normal University, Changchun, China; 2 School of Management, Changchun University of Finance and Economics, Changchun, China; Institute for Advanced Sustainability Studies, GERMANY

## Abstract

**Background:**

Previous studies indicate that migrant integration is associated with migrants’ characteristics as well as restrictions and opportunities in receiving cities. However, the effect of receiving cities and the relationship between migrants and receiving cities have not been fully explored due to the lack of large samples from cities. The objective of this study is to examine the effects of receiving cities alone and their regulating role in the interaction with individual characteristics.

**Methods:**

Cross-city data on 154,044 Chinese domestic migrants above 15 years old in 289 cities from the 2017 China Migrants Dynamic Survey are used. Migrant integration is assessed by a four-dimensional model proposed by Esser, which is slightly adjusted according to the characteristics of Chinese migrants. A hierarchical linear model is used to measure the proportion of effects of city factors in migrant integration as well as the effects when city factors are considered alone and in interaction with individual factors.

**Results:**

The individual-level and city-level factors are responsible for 69.81% and 30.19% of the effect on migrant integration, respectively. City political factors do not affect migrant integration directly, and cities with larger sizes and higher wages can directly and significantly improve integration, while higher housing prices will directly inhibit integration. From the cross-level interaction of city and individual, different social, economic and political factors at the city level have an indirect impact on migrant integration by inhibiting or strengthening the effect of individual-level factors on migrant integration.

**Conclusion:**

This study is one of the first to show the effect of cities and the relationship between receiving cities and migrants on migrant integration by keeping the national context constant. It is necessary to weaken the social and economic privileges associated with a city’s administrative level and reduce the negative impact of cities’ social and economic conditions by implementing city agglomeration, developing advantageous industries and optimizing the industrial structure. It is also essential to improve migrants’ socioeconomic capital through social support, occupation training and contiguous education.

## Introduction

The large-scale integration of domestic migrants has become an important problem for Chinese urbanization in recent years. The rate of urbanization in China currently has exceeded 50%. However, urbanization is not the only process of population agglomeration in cities through migration from rural areas; the transformation of modes of production and life through citizenization has also contributed [1, 2]. At present, Chinese domestic migrants have completed the process of urbanization in the areas in which they reside but have yet to be completely citizenized in economic, social, and cultural terms. This state of affairs indicates that China’s urbanization is incomplete and low quality, and migrants are excluded from full participation in the receiving cities to some extent [3]. Therefore, there is an urgent need to explore migrant integration to promote high-quality urbanization in China and achieve sustainable internal migration.

In the context of the concern about migrant integration, literature and policy debates have often focused on the incompatibility of individual characteristics, including narrow social networks and human capital [4], limited social participation in neighborhoods [5], as well as institutional restrictions such as the national household registration (Hukou) system [6]. In addition to these factors, the characteristics of the receiving city are also important in this context because they are antecedent conditions for migrants [7]. However, these relevant findings default to the premise that the locations within which migrants try to integrate are almost uniform [8–10]. The cities’ features have not been fully addressed in the literature. Therefore, the goal of this article is to empirically explore the contextual roles of socioeconomic conditions, demographic composition and institutions of receiving cities on the integration of migrants based on a large sample.

In a context in which large-scale domestic migration has become widespread, the integration of migrants in receiving cities has become a problem that all cities need to pay close attention to. Specific corresponding countermeasures must be put forward by receiving cities according to their unique characteristics. Therefore, it is of great practical value to study the interaction between migrants and receiving cities. Specifically, this paper explores this issue from three aspects innovatively. (I) The proportion of the effects of individual-level and city-level factors is distinguished. (II) The effects of city-level factors and individual-level factors are examined separately, and the cross-level effects of migrants and cities are also measured. (III) The application of large-sample data can make the empirical analysis more stable and reliable and provide guidance for cities in China.

The rest of this study is organized in five sections. Section 2 presents a brief literature review. Section 3 introduces the data sources and methodology. Section 4 presents the main findings. Section 5 discusses the findings and provides suggestions. Section 6 highlights the conclusions.

## Literature review

### Definition and measurement of migrant integration

The definition and concept of migrant integration were not particularly clear in early studies due to conceptual arbitrariness in the social science on the one hand and scientific controversies on the other hand. The adaptation, acculturation, assimilation and incorporation emerged [11]. Adaptation, assimilation and acculturation are used to describe the unidirectional acceptance of the receiving place, the process of eliminating differences, and the acquisition of the receiving place’s language and culture, respectively [12, 13]. With the deepening of the research, the terms integration and incorporation are used and focused mostly on migrants of the second generation. These terms are used as neutral, superordinate concepts that refer in a general way to the (interdependent) relations between persons (or groups) [11], and adaptation, assimilation and acculturation are thought to be potential forms of incorporation or integration. Compared with incorporation, the term integration is better suited since it is compatible with general sociological theory. This paper applies the concept of migrant integration to describe a state of equal interaction and acceptance between migrants and receiving places, including locals.

There are few detailed and empirical measurements of migrant integration in models whose subjects are immigrants before 1965, and migrant integration is described qualitatively [14, 15]. ① Race relation models put forward a four-stage model and seven-stage model to assess assimilation. The former includes contact, conflict, accommodation, and assimilation [16], and the latter includes curiosity, economic welcome, industrial and social antagonism, legislative antagonism, fair-play tendencies, quiescence, and second-generation difficulties [17]. This models depict the process of migrant integration as progressive and irreversible, with only one possible endpoint: the complete assimilation of the migrant group [18, 19]. ② The social-psychological model analyzes the change in group membership from the perspective of the individual and emphasizes attitudes, norms, role attitudes, and role behaviors [20]. This measurement of migrant integration is more detailed and operational but not sufficiently systematic. ③ The multidimensional assimilation model proposed by Gordon represents a milestone in the study of migrant integration. This model includes cultural or behavioral, structural, marital, identificational, attitude receptional, behavioral receptional, and civic assimilation [21]. Complete assimilation is thus not necessarily the endpoint of the integration of ethnic minorities, and stable forms of ethnic stratification have emerged [21].

To date, the multidimensional measurement of migrant integration has basically formed and guided contemporary models. The model of intergenerational integration distinguishes four dimensions of integration: the cultural dimension, which refers to the acquisition of skills and knowledge of language, customs and lifestyle; the structural dimension, which is the core of participation in the labor market and occupation; the social dimension, which refers to the interaction and contact with autochthonous people; and the emotional dimension, which concerns identity and belonging [22]. The social and systematic model considers migrant integration according to three interdependent aspects: individual social integration, which describes migrants’ inclusion into or exclusion from social (sub)systems; social inequality and social differentiation between groups; and societal integration as a whole, which refers to the relationships between different social systems [23]. The new immigrant geographical models suggest the investigation of the unequally configured geographies of these newer immigrant locations and of how internal migration interacts with wage inequality more generally. These models provide an opportunity to move such discussions beyond the theoretical and empirical constraints of migration selectivity and spatial assimilation models [24, 25].

### Influencing mechanisms of migrant integration

The research on the influence mechanisms of migrant integration has experienced a change from focusing on individual characteristics to focusing on both the individual and the context.

The early models focus on the characteristics of the individual as well as the interaction between individuals. The differences in language, religion, race and culture between migrants and locals are the objective basis for the integration. These differences bring about inevitable conflict and potential integration for migrants and locals. Competition between individuals in valued economic and social conditions are first considered as the driving force of conflicts and assimilation [26], and the resulting dynamic relationship between individuals and locals has begun to gain attention. On this basis, the role of the individual receives a more detailed analysis. Individual characteristics such as intelligence, tolerance, adaptability and talents can help migrants optimize internal and external group membership [20, 26].

The receiving society’s characteristics have begun to be mentioned with the deepening study of migrant integration. With the extension of the residence time of the migrants in the receiving locations, migrant integration differs in different places, and the role of the environment has gradually been recognized. However, as with individual factors, the emphasis is on the context’s various attitudes towards migrants, which can vary among “pressing, willing, indifferent, unwilling or blocking” and may cause different states of migrant integration: monistic, pluralistic, and interactionist [26]. The focus on individual immigrants’ motivation and actions and their adjustment to their social environment has advanced the study of migrant integration.

Both individual and environmental factors and their interplay have been considered in depth with the important contribution and influence of immigrants to the receiving place. The interplay of migrants’ motives and skills and the receiving society’s opportunities and restrictions influences migrant integration [22, 27–30]. At the individual level, human, social and ethnic capital; motivation; and cognition are essential factors [31–34]. Migrants can decide to invest energy and resources into receiving places to bring about the best integration results. At the contextual level, restrictions and opportunities are the central factors that constitute the structural frame for individual action, in particular, the (ethnic) community migrants are embedded in and the relevant segment of the receiving society they can acculturate to [23, 35, 36]. It is clear that the influencing mechanisms of migrant integration change with migrants’ characteristics and the social and economic environment, and the influencing mechanisms should be comprehensively considered from these two aspects.

### Studies of migrant integration in China

Even if there are nuanced descriptions and explanations of migrant integration, this field remains fragmented, and the applicability of existing models in China remains to be examined. First, the study objectives are different. Existing models are generally based on transnational migrants with different ethnic and racial backgrounds. China has a large land area and a large number of domestic migrants, and they mostly share the same ethnic, racial and unified Chinese culture and official language among themselves and with the local people. This means that there will be no serious ethnic, racial or cultural discrimination and conflict. Second, the economic system is different. China has a market economy with socialist characteristics, while most other countries have a market economy [37]. As a result, China’s migrants and local people have economic competition that is not polarized under the control of the Chinese government, reducing employment discrimination and avoiding serious residential segregation and community differences [38]. Third, migrants’ needs for integration are different. Cultural, residential, and structural integration are more important for migrants in other countries, where the public resources are relatively fair and rich among different regions. However, the spatial distribution of public welfare is unbalanced in China. Therefore, fair access to public opportunities and welfare is more important for Chinese domestic migrants than permanent migrants in other countries [39, 40].

Referring to foreign studies and characteristics of Chinese domestic migrants, Chinese scholars usually define migrant integration from the perspective of social equity. Migrant integration refers to migrants gradually gaining basic economic and social insurance, accepting or adapting to the subculture of receiving cities, and engaging positive interactions with the local population on the basis of equal treatment between migrants and the established population [41, 42]. Generally, Chinese scholars have studied migrant integration in China’s specific environment and formed four research paradigms. First, the demographic paradigm holds that migrant integration is affected by the individual’s human capital. A lack of essential education and job qualifications can lead to failure to meet labor market demands and a low degree of integration [43]. Second, the social paradigm holds that the extreme scarcity of social capital leads to limited access to economic, cultural and political capital, thereby hampering migrants’ integration ability [44]. Third, the institutional paradigm tends to regard the unique social institutional structure, including household registration, education and other social management systems, as a determinant of whether migrants can achieve integration. Especially in China, migrants with agricultural Hukou may experience invisible discrimination that slows their process of integration [45, 46]. Fourth, the geographical paradigm addresses the impact of city-specific factors on the integration of Chinese migrant workers. The roles of dialect, city size, employment opportunities and education in receiving cities have been evaluated [9]. Generally, both migrant factors and unique macroinstitutional environmental or microcity features have an important impact on migrant integration.

**Fig 1:**
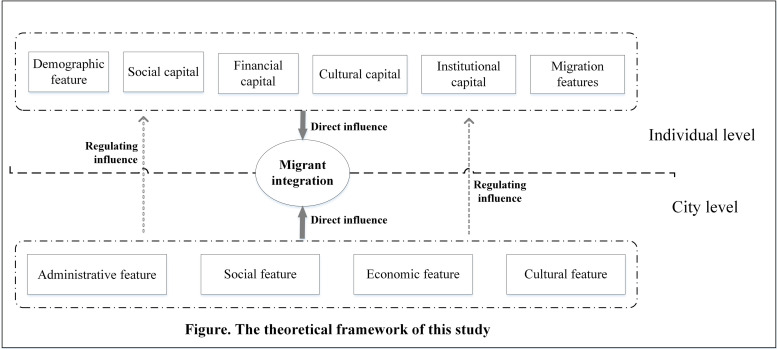
The theoretical framework of this study.

Summarizing previous research results, the important roles of both individual characteristics and city context in migrant integration have been proven. However, there are still two limitations that hinder further studies of Chinese domestic migrants. The first is the foundation of research, in which the utilization of survey data from a single city or several cities in one province has prevented scholars from examining city effects on migrant integration while keeping the national context constant [47]. Second, although studies offer insights about relevant city-specific factors affecting migrant integration, the interaction of city-specific factors and individual-specific factors has not been fully clarified. This paper extends the spatial scope to cities across China and, more importantly, creates a multilevel approach that combines both individual-level and city-level factors to treat humans and places as an integrated and interactive system (Fig 1).

## Materials and methods

### Data sources

The empirical analysis in this paper is based on the China Migrants Dynamic Survey (CMDS) conducted in 2017, which includes 289 cities and 154,044 Chinese domestic migrants over 15 years old who live in the receiving city for more than one month. The survey is conducted by the National Health Commission with the layered, multistage and probability proportionate to size (PPS) sampling method, and the total migrants in 2016 is the basic sampling frame. The sample cities account for 82.86% of all Chinese cities. Only those cities with poor natural conditions and very few migrants were not selected for the questionnaire survey. In this survey, a city is defined as a built-up area with urban functions, and Chinese domestic migrants are defined as people who have a Chinese household registration and migrate across the boundaries of county or higher administrative level in China. The data on city features are obtained from China’s City Statistical Yearbook 2018.

### Methodology specification: Hierarchical linear model

A hierarchical linear model (HLM) is used to model how integration varies across individuals and cities and can be considered a generalization of the ordinary least squares regression when the dependent variable varies at more than one level [48, 49]. Using HLM 6.08 software, we first establish a basic model, called Model 0, in which there are no variables at either level. Model 0 is as follows:

$$\text{Level-}1\;\text{model}:\;Y_{ij}=\beta_{0j}+r_{ij}$$ (1)

$$\text{Level-}2\;\text{model}:\;\beta_{0j}=\gamma_{00}+\mu_{oj}$$

If the variance components pass the examination of significance, the dependent variables are affected by both level 1 and level 2. Therefore, two semiconditional models should be established. One model is a single first-level independent variable model with no variables at the second level (model 1). Model 1 is as follows:

$$\text{Level-}1\;\text{model}:\;Y_{ij}=\beta_{0j}+\sum \beta_{nj}X_{nij}+r_{ij}$$ (2)

$$\text{Level-}2\;\text{model}:\;\beta_{0j}=\gamma_{00}+r\mu_{oj}$$

$$\beta_{nj}=\gamma_{n0}+\mu_{oj}$$

The other model is a single second-level independent variable model with no variables at the first level (model 2). Model 2 is as follows:

$$\text{Level-}1\;\text{model}:\;Y_{ij}=\beta_{0j}+r_{ij}$$ (3)

$$\text{Level-}2\;\text{model}:\;Y_{ij}=\beta_{0j}+\sum \gamma_{0m}W_{mj}+r\mu_{0j}$$

If the variance of the intercept and slope ratios at the first level is significant at the second level, establishing that a model covering the variables at both levels (model 3) is necessary. Model 3 is as follows:

$$\text{Level-}1\;\text{model}:\;\beta_{0j}=\gamma_{00}+\sum \beta_{nj}X_{nij}+r_{ij}$$ (4)

$$\text{Level-}2\;\text{model}:\;\beta_{0j}=\gamma_{00}+\sum \gamma_{0m}W_{mj}+r\mu_{0j}$$

$$\beta_{nj}=\gamma_{n0}+\sum \gamma_{nm}W_{mj}+\mu_{nj}$$

where i is individuals; j is a city; X is the variables at the individual level; *Y*_*ij*_ is the degree of integration; *β*_0*j*_ is the intercept of city j; *β*_*nj*_ is the slope of variable *X*_*n*_ in city j; *r*_*ij*_ is the residual of individual i in city j; *γ*_00_ and *γ*_*n*0_ are intercepts; and *γ*_0*m*_ and *γ*_*nm*_ are the slopes predicting *β*_0*J*_ and *β*_*nj*_ from variable *W*_*m*_ at the second level, respectively.

### Selection of variables

#### Explained variable: Migrant integration

Under the framework of a relatively mature and comprehensive four-dimensional measurement of migrant integration in the intergenerational model proposed by Esser, the selection and definition of some indicators are slightly adjusted according to the characteristics of Chinese domestic migrants. The four dimensions are cultural, structural, social and emotional integration (Table 1). For the cultural dimension, due to the consistency of Chinese traditional culture and language in different regions, only the acceptance of migrants into receiving cities’ living habits and subcustoms is investigated. For the structural dimension, migrants’ economic status is still the central indicator of the intergenerational model. Personal income and labor contract stability are selected to measure economic level and stability. On the one hand, the social dimension examines the interaction between migrants by checking the frequency of activities in which migrants participate, and on the other hand, social integration is examined by checking whether migrants have social insurance and residence rights, which are crucially important in China’s population management system. The emotional dimension is different from that in foreign countries in that discrimination and prejudice are almost nonexistent. Therefore, this paper only investigates the recognition of migrants in the receiving city.

**Table 1:** Index system of migrant integration and its summary statistics.

Indicator	Description	Value	Mean	S.E.
**Cultural dimension**				
Customs/habits	“It’s not important to follow the customs and habits of my hometown.”	1 = completely disagree, 2 = disagree, 3 = basically agree, 4 = fully agree	2.41	0.83
**Structural dimension**				
Personal income	“What was your personal income last month?”	Logarithm of income	2.19	1.12
Labor contract	“Which type of contract have you signed?"	1 = no labor contract, 2 = a one-time work position or internship, 3 = labor contract is signed, but there is no fixed term, 4 = a formal labor contract	2.60	1.25
**Social dimension**				
Social participation	“Have you made donations or engaged in other voluntary activities in this city?”	1 = never, 2 = rarely, 3 = sometimes, 4 = often	1.49	0.75
Social insurance	“Do you have a social insurance card?”	1 = not applicable or I do not know about it; 3 = I know about it but haven’t processed it; 4 = I have processed a card	2.25	0.91
Residence rights	“Do you have a residence rights card?”		3.16	1.06
**Emotional dimension**				
Identification	“I feel that I am already a local.”	Same as “customs/habits”	2.96	0.76

The index system of migrant integration is standardized and used for the factor analysis. On this basis, the degree of migrant integration is calculated by taking the variance contribution rate after rotation of the three new factors whose cumulative contribution is over 86% as the weight (Table 2) and converting it to a value of 0–100 according to the standard score.

**Table 2:** Results of the factor analysis of migrant integration (rotating factor matrix).

Index	Factor 1	Factor 2	Factor 3
**Social insurance**	0.958	0.038	0.029
**Residence rights**	0.954	0.043	0.032
**Personal income**	0.049	0.761	0.021
**Labor contract**	0.171	0.741	0.056
**Identification**	0.025	-0.207	0.701
**Customs/habits**	0.019	0.058	0.690
**Social participation**	0.109	0.190	0.621

### Explanatory variables at level 1: Individual-level variables

Previous studies have proven that migrants’ characteristics, ability and capital are determinant factors. Therefore, migrants’ demographic and migration characteristics are first examined. Migrants’ demographic characteristics are measured by gender and age. Behind the age difference of migrants is the intergenerational difference of migrants, and to compare the differences in social integration among the young, the middle-aged and the elderly, we divide age into three categories according to the measurement of the United Nations: 14–45, 46–60 and > 60. In addition, the residence length is selected to describe migrants’ migration status. Moreover, based on the field-capital-habitus framework of sociology [50], variables reflecting migrants’ social, financial, and cultural capital are collected. Social capital refers to resources that individuals may receive from social networks and the social systems in which they live [51]. This study uses “families’ company”, “hometown’s fetters”, and “local support” to reflect the migrant social capital obtained from families, hometowns and receiving cities, respectively. Financial capital is equivalent to the concept of capital in the sense of general economics, and we use “occupation” to reflect migrants’ pursuit of their financial interests (details on the classification of occupations are shown in S1 Appendix). Cultural capital is the sum of an individual’s knowledge, technology, temperament and cultural background based on the possession of cultural resources. This paper uses the foundational item of “education” to reflect the possession of cultural capital by migrants [44, 52]. In addition to the above capital, it is thought that the capital related to the governmental management of migrants is also important to Chinese migrants based on China’s population management system, and this factor is named institutional capital. Institutional capital potentially affects migrants’ access to other types of capital. The details of variables are shown in Table 3.

**Table 3:** Sample statistics of individual-level variables (n = 154,044).

Categorical variable	Categories	Sample size	Proportion
**Demographic characteristic**			
Gender	Female	74729	48.51
	Male	79315	51.49
Age	15–45 years old	118022	73.37
	46–60 years old	29580	19.20
	>60 years old	5566	3.61
**Social capital**			
Families’ company	Migrates alone	37367	24.26
	Migrates with family	116677	75.74
Hometown’s fetters	There are no fetters from the hometown	60304	39.15
	There are fetters from the hometown such as caring for the ill and elderly, spouse living alone and others	93740	60.85
Local support	Migrant receives help in receiving cities	83906	54.47
	Migrant does not receive help in receiving cities	70138	45.53
**Financial capital**			
Occupation	In secondary labor market	109060	70.80
	In primary labor market	44984	29.20
**Institutional capital**			
Hukou	Agriculture Hukou	119341	77.47
	Nonagricultural Hukou	34703	22.53
Continuous variable	Definition	Range	Mean (SD)
**Cultural capital**			
Education	Years of school education	0–17	9.85 (2.87)
**Migration characteristic**			
Residence time	The time migrants live in the receiving city	0.08–69.17	6.47 (6.00)

### Explanatory variables at level 2: City-level variables

The cities’ contextual characteristics are proven to have fundamental effects on migrant integration. Therefore, cities’ political, social, economic and cultural statuses are examined to understand the unique background of receiving cities (Table 4). First, cities’ administrative level is selected to reflect their different capacities to control social resources under the national political system and is categorized into four groups based on the regulations of the Chinese government: common prefecture city, ordinary capital city, planned/subprovincial city, and municipality. Considering the small number of the latter three categories of city, this paper divides cities into two categories: low administrative level cities, which mainly refers to ordinary prefecture-level cities, and high administrative level cities, mainly refers to ordinary capital cities, planned/subprovincial cities, and municipalities. Second, education, health care and other social benefits are usually allocated according to the population of a city, so the variable for cities’ social characteristics is computed as the resident population of receiving cities [53]. According to China’s criteria for city size, city size is divided into five categories to reflect the richness of social resources: small city (population <500,000), medium city (500,000–1,000,000), large city (1,000,000–5,000,000), mega city (5,000,000–10,000,000) and super city (>10,000,000). Similar to the administrative level, city size is also divided into two categories to identify its role more clearly: one is smaller cities, including small and medium-sized cities; the other is larger cities, including large, mega and super cities. Third, approximately 80% of Chinese domestic migrants migrate for higher economic benefits, and the purchase of housing is a symbol of the connection between a person and the place. Therefore, three variables are used as proxies to carefully examine cities’ economic conditions: the gross domestic product (GDP) per capita to measure the overall level of economy, the per capita wage to measure the level of economic acquisition, and the housing price to measure economic expenditure.

**Table 4:** Sample statistics of city-level variables (n = 289).

Categorical variable	Classification/definition	Sample size	Proportion
**Political status**			
Administrative level	Low administrative level city	253	87.54
	High administrative level city	36	12.46
**Social status**			
Size	Smaller city (population <1,000,000)	189	65.40
	Larger city (population >1,000,000)	100	34.60
Continuous variable	Range	Mean	SD
**Economic status**			
Per capita GDP, yuan	11,892–215,488	53569.98	31027.44
Per capita wage, yuan	36,793–122,749	54443.36	7935.78
Housing price, yuan	2,458–57,768	7790.75	6621.65

## Results

### Score of migrant integration and model estimation

The score of migrant integration is calculated through factor analysis. For the individual level, the average score of migrant interaction is 52.30, the range is 16.27–84.67, and the standard error is 13.44, showing that migrant integration is generally at a medium level and that there are substantial differences among migrants. For the city level, the values of the above three indicators are 51.19, 31.61–73.11, 9.34, indicating that the gap of migrant integration is also substantial among cities. From the region level, there are greater differences among the four regions of China. The central region has the highest score of 60.54 in migrant integration. The west and northeast regions follow, with scores of 57.80 and 54.55, respectively. The east region has the lowest score of migrant integration of 45.11. Although more Chinese domestic migrants migrate to the developed east region, their integration is not advanced.

Model 0, which passed the examination of the significance of intergroup correlation coefficients (ICC>0.059) and chi-square values (*p* = 0.000, df = 288), indicates that migrant integration is affected not only by personal characteristics but also by receiving cities’ characteristics. The individual-level and city-level factors are responsible for 60.81% and 30.19% of the effect on migrant integration, respectively, indicating that the effects of neither individual nor receiving city characteristics can be ignored. Therefore, a hierarchical model is needed to analyze the impacts of these two levels.

### The direct effects of variables at the individual and city levels on migrant integration

#### The direct effects of individual-level variables on migrant integration

The results of Model 1 are shown in Table 5, and the direct effects of individual-level variables on migrant integration are measured. The effects of each kind of capital on integration are different, and the internal functions of each capital or migrant’s characteristics are not completely consistent. (a) Regarding migrants’ characteristics, inherent demographic differences make a significant difference in the degree of integration. Men are more likely to integrate into receiving cities, and this is closely related to the economic weakness of women. More than 90% of women who do not work are pregnant or taking care of families, and women with jobs earn an average of 10% less than men. For different stages of life, compared with young migrants, middle-aged migrants are more likely to integrate into receiving cities, while elderly migrants are less likely to integrate. The disadvantages of elderly migrants with respect to integration are strengthened by the gradual weakening of physical fitness and working stamina. In addition, this positive effect of residence time shows that migrant integration is gradually improved with the continuous improvement of residence time. (b) Some forms of social capital do not necessarily promote integration, such as families’ company and hometown’s fetters. Although families might promote psychological stability for migrants, the diverse structures and different abilities of families lead to different statuses in terms of integration. Similarly, sending cities not only give migrants an economic "push" but also have a psychological "pull" at the same time. Some social capital will have significant effects on integration. For example, local support significantly increases the possibility of integrating into receiving cities. (c) The effects of financial and cultural factors are constant. With gains in financial and cultural capital, the degree of migrant integration is likely to increase. The gaps between occupations in the two labor markets and in education across years are significant. On the one hand, the important role of financial and cultural capital is proven, and their roles in improving integration are clear. On the other hand, the fact that most migrants have jobs with low wages and low security, as well as less education or job skills training, poses a huge challenge to integration. (d) For institutional capital with Chinese characteristics, agricultural Hukou will significantly decrease the degree of integration through long-term disadvantages created by the traditional population management system.

**Table 5:** Analysis results of model 0.

Hierarchy	Variance component	Contribution ratio	Degree of freedom	Chi-square	*p*
Level-2	5.31	30.19%	288	29691.11	***
Level-1	12.28	69.81%			

***, *p*<0.01.

#### The direct effect of city-level variables on migrant integration

The results of Model 2 are shown in Table 6 and demonstrate that city-level factors directly affect migrant integration mainly through receiving cities’ social and economic features but not their institutional or cultural characteristics. (a) Changes in city administration level will not have an impact on integration. This means that the political advantages of a city and the unbalanced resources favoring high administrative levels do not necessarily promote integration. This discrepancy can be attributed to the unity of Chinese decrees. In other words, although different provinces and different cities take different measures in the policy practice of migrant management, the principles of these policies are the same. (b) Compared with smaller cities, larger cities are more likely to promote integration, indicating that only when the city reaches a certain scale will the positive effects of population agglomeration appear. The larger the city size, the greater the promoting effects on migrant integration are. This effect occurs because the expansion of city size is accompanied by various scale effects that provide better social conditions, allowing every migrant to give full play to his or her own strengths to achieve integration. (c) The effects of the three economic factors on integration are different. Per capita GDP has no impact on migrant integration, while the per capita wage and housing prices significantly affect migrant integration. This condition indicates that economic factors closely related to migrants’ salary are more influential than cities’ general economic status. Therefore, the higher the per capita wage and the lower the housing costs are, the higher the integration of migrants. The dual role of these two economic factors puts migrants in a dilemma and forces migrants to find a balance in receiving cities.

**Table 6:** Effect of individual-level and city-level factors on migrant integration.

Individual-level variable	Coefficient	*P*	City-level variable	Coefficient	*P*
Intercept	36.57	***	Intercept	43.19	***
Gender (ref: female)			Administrative level (ref: low administrative level city)		
Male	2.59	***	High administrative level city	-2.10	0.47
Age (ref: 15–45 years old)			Size (ref: smaller city)		
46–60 years old	0.80	***	Larger city	3.52	**
>60 years old	-3.43	***	Per capita GDP	-0.31	0.71
Residence time	1.48	***	Housing price	3.21	**
Families’ company (ref: Migrate alone)			Per capita wage level	3.70	***
Migrate with family	-0.01	0.90			
Problems in hometown (ref: no)					
Yes	0.07	0.50			
Social support (ref: no)					
Yes	1.54	***			
Occupation (ref: secondary labor market)					
Primary labor market	3.68	***			
Education	1.24	***			
Hukou (ref: nonagricultural Hukou)					
Agricultural Hukou	-0.39	***			

**, *p*<0.05;

***, *p*<0.01.

### Cross-level interaction of city-level variables on the effect of individual-level variables

Since integration is the process of the interaction between migrants and receiving cities, the regulatory effect of a city’s environment on individual characteristics will affect the realization of integration, as revealed by model 3. The significant regulatory effects of city-level factors on individual-level factors are shown in Table 7. Four regulatory mechanisms by which city-level variables strengthen or weaken the original function of individual-level variables are listed in Table 7.

**Table 7:** Cross-level interaction of city-level factors on the effect of individual-level factors.

Cross-level effect	Migrant integration		Regulatory mechanism
	Value	P	
Occupation (ref: secondary labor market)			
City size	-1.90	***	-+
Per capita GDP	2.81	***	++
Housing price	1.68	*	++
Per capita wage	-1.08	***	-+
Age (ref: 15–45 years old)			
46–60 years old			
Administrative level	-1.30	**	-+
Per capita GDP	1.10	***	++
Per capita wage	0.79	**	++
>60 years old			
Administrative level	-1.53	**	--
City size	-3.59	***	--
Per capita GDP	-0.49	**	--
Housing price	-2.86	***	--
Education			
City size	0.33	***	++
Per capita GDP	0.49	***	++
Housing price	0.68	***	++
Residence time			
Administrative level	-1.91	*	-+
City size	0.06	**	++
Hukou (ref: nonagricultural Hukou = 0)			
Administrative level	-0.63	*	—
City size	1.33	***	+-

“+ +”: city-level variables strengthen the positive effect of individual-level variables; “− +”: city-level variables weaken the positive effect of individual-level variables; “+ −”: city-level variables weaken the negative effect of individual-level variables; “− −": city-level variables strengthen the negative effect of individual-level variables.

a. Insignificant coefficients are not reported in this table.

b. The setting of the reference variables is the same as in Table 6.

c. **, *p*<0.05; ***, *p*<0.01.

The first regulatory mechanism is that city-level variables strengthen the positive effect of individual-level variables, causing individual-level variables to play a stronger role in integration in specific city contexts. In cities with higher per capita GDP and housing price, migrants in the primary labor market and those with higher education able to integrate more easily. With better occupations and education, professional and technical migrants obtain access to the core resources of these cities. In large cities, the positive effects of education and residence time on migrant integration are stronger. Knowledge spillover and agglomeration effects are stronger in these cities, making the rate of return on education and socioeconomic accumulation higher than in other cities, directly promoting the positive effects on migrant integration. In cities with higher capital wages and GDP, middle-aged migrants who both have richer working experience and stronger working ability are more easily to integrate into receiving cities.

The second regulatory mechanism is that city-level variables weaken the positive effect of individual-level variables. That is, the positive effect of city-level variables is not as strong as it used to be in specific city contexts. In cities with larger size and higher capital wage, the positive effect of occupations in the primary labor market on integration is weaker. This effect indicates that every occupation, not several dominant occupations, has received sufficient attention in these cities, and the differences among different occupations are reduced. In cities with higher administrative level or per capita GDP, the advantages to the integration of middle-aged migrants and longer residence time are weakened. In cities with higher administrative level, the advantages of middle-aged migrants in work experience could hardly cover the restrictions brought by high administrative level. Moreover, it is difficult to achieve these benefits over time.

The third regulatory mechanism is that city-level variables weaken the negative effect of individual-level variables. That is, the original negative effects of individual-level variables on migrant integration become weaker. The most notable effect is that the negative effect of agricultural Hukou on migrant integration is further reduced in larger cities since these cities can provide rich job opportunities. Additionally, these cities have more developed service industries, which also makes employment more accessible and fairer. Therefore, every migrant can find a suitable job, which makes it easier to achieve a basic economic foothold and lays the foundation for further integration. Coupled with the reduction in the demographic dividend in China, as long as migrants work hard, they are more likely to be paid relatively generously. This can explain why most migrant workers face many obstacles when integrating into large cities, but they still migrate to these cities on a large scale.

The fourth regulatory mechanism is that city-level variables strengthen the negative effect of individual-level variables, strengthening the original negative effects of individual-level variables on migrant integration. This regulatory mechanism mainly works on the elderly migrants and migrants with agricultural Hukou. For the elderly migrants, their disadvantage in the integration has been further amplified in cities with higher administrative level, per capita GDP, housing price and larger city size. This effect is caused by the mismatch between the characteristics of the city and the elderly. On one hand, economic development is more rapid in more developed cities. On the other hand, the elderly have a weakened ability to learn new things. Therefore, the implementation of social assistance from families, society and government is needed. For migrants with agricultural Hukou, their disadvantages are magnified in cities with a high administrative level, where there are usually strict restrictions on Hukou conversion. This result suggests that local urbanization is a feasible way to realize integration.

## Discussion: Policy implications

### Migrant integration and cities’ administrative governance

This paper confirms that traditional Hukou policies and high administrative level significantly slow migrant integration. Although a city’s administrative level has no direct and significant impact on integration, it indirectly affects migrant integration through a regulatory mechanism. Our results suggest that in cities with a high administrative level, it is more difficult for middle-aged and elderly migrants, migrants with agricultural Hukou and those in the primary labor market to integrate. Despite the recognition and pride associated with a higher administrative level that regulates Chinese people’s political and daily lives, the expectations of migrants regarding their political status are not met in these cities. More deeply, administrative differences usually translate to differences in the social, economic and political environment [39]. Therefore, this paper suggests peeling away the socioeconomic policy privileges attached to the administrative hierarchy and maximizing fairness in migrant policy between cities of different administrative levels. On this basis, different administrative levels can become an effective policy means for the management of migrants rather than barriers weakening the capital of vulnerable groups.

### Migrant integration and cities’ economic conditions

This paper partially confirms previous results indicating that the per capita GDP and housing costs of a receiving city negatively affect migrant integration [9]. We find that housing prices negatively affect migrant integration, that per capita GDP has no significant effect based on national survey data, and that a city’s wage level has a positive effect on migrant integration. These differences can be attributed to the stability of a model based on a large sample. In addition, the gap between primary and secondary labor market increases in cities with higher per capita GDP and housing prices. Moreover, it is more difficult for middle-aged, and especially elderly, migrants to integrate into more developed or larger receiving cities. Therefore, the first step to optimize migrant integration is to eliminate the social and economic rights and interests attached to housing and to regulate housing prices. The second step is to adjust the economic structure to avoid polarization between the two labor markets. The third step is to provide more assistance to vulnerable migrants to lay a stable foundation for economic development.

### Migrant integration and cities’ social conditions

Our results confirm that from the perspective of the city alone, larger cities provide multiple potential employment opportunities for migrant integration, promoting economic integration and leading to psychological estrangement. From the perspective of the regulatory mechanism of city size, the positive roles of education and residence time are enlarged and negative role of agricultural Hukou is narrowed. This explains why even if cities’ conditions are excellent, the integration of most migrants with low academic qualifications is not optimal. Even so, migrants continue to head for larger cities because these cities provide the best opportunities for individuals. Therefore, directly encouraging migrants to move to small and medium-sized cities to realize integration at a low cost would not be effective. Thus, an urban pattern of coordinated development among small, medium, and large-sized cities and small towns should be encouraged. This would not only alleviate the decline in population agglomeration capacity and the inadequacy of public services in larger cities but also promote the development of smaller cities.

### Migrant integration and individuals’ socioeconomic capital

In this paper, we show that enhancement of social and economic capital is helpful in compensating for a shortage in migrant integration and that migrants can take the initiative to learn to obtain this capital. On one hand, the role of social capital is mainly reflected in social assistance to migrants. Therefore, migrants can actively seek help from local people and local government when they encounter problems. Communities also need to offer comprehensive and essential support for migrants in daily life. This interaction promotes the relationship between migrants and receiving cities, laying a foundation for the further integration. On the other hand, the role of economic capital is mainly reflected in occupation. The impact of occupation improvement on integration is the most obvious. Vocational skill trainings are urgently needed, which can lay the foundation for migrant integration in the short term. Education can promote both migrants’ social and economic capital. The role of education is long term and invisible. So, the promotion of continuous education after graduation is also needed to make full use of the positive effect of individuals’ capital.

### Migrant integration and future challenges

Three challenges are summarized in this paper and have a fundamental impact on migrant integration. The first is the aging of migrants. Migrants’ disadvantages are substantial and are magnified in cities with better social and economic conditions. This problem needs to be addressed immediately; otherwise, it will cause serious social problems. The basic task is welfare planning for elderly migrants, such as providing public nursing home beds. The second challenge is the excessive concentration of migrants in specific super cities. Based on the respect and protection of the rights and wellness of migrants, a rational distribution of migration may be realized through regional coordinated development to balance the social and economic resources among different cities. The most important task is to promote rational migration by developing city agglomeration to achieve the balance of individual capital and city features to smooth the road to assimilation. The third challenge is the urbanization of the agricultural population. The opportunities of migrants with agricultural household registration coexist with and restrictions in integration. Agricultural migrants will take a long time, even several generations, to realize high urbanization and citizenization. Therefore, promoting the fairness of the agricultural migrants and others in education, housing and employment is a long-term work.

### Conclusion

Through a survey of migrants in China, this paper develops an index system to measure migrant integration, and the degree of migrant integration is low. The hierarchical linear model indicates that individual-level and city-level factors are responsible for 70.52% and 29.48% of the effect on migrant integration, respectively. From the perspective of individuals, well-educated, male, middle-aged migrants and those with nonagricultural Hukou, a longer residence duration, more social support and employment in the primary labor market are more likely to integrate into receiving cities. From the perspective of receiving cities, cities with larger sizes and higher wages can directly and significantly improve integration, while higher housing prices will directly inhibit integration. From the cross-level interaction of both city and individual levels, different city-level social, economic and political factors have an indirect impact on migrant integration by inhibiting or strengthening the effect of individual-level factors on migrant integration. It is necessary to weaken the social and economic privileges associated with a city’s administrative level and reduce the negative impact of cities’ social and economic conditions by implementing city agglomeration, developing advantageous industries and optimizing the industrial structure.

Due to length limitation, this paper examines only the interaction between city characteristics and migrant integration as a whole. In the future, we will explore the dimensions of migrant integration and consider which dimension is affected by what factors—especially in terms of the regulating influence of city-level variables on individual-level variables in the context of integration—to obtain a more thorough understanding of the roles of individuals and cities and to provide more targeted suggestions to promote cities’ urbanization and sustainability.

## Supporting information

S1 Appendix Classification of occupations. (DOCX)
