# Supplementary material for: How do city-specific factors affect migrant integration in China? A study based on a hierarchical linear model of migrants and cities
Source: PLoS One. 2021 Jan 12;16(1):e0244665. doi: 10.1371/journal.pone.0244665 (PMC7802925; doi:10.1371/journal.pone.0244665)
Supplement: S1 Appendix — (DOCX) [file pone.0244665.s001.docx]

**S1 Appendix. Classification of occupations**

| Classification | Occupation |
| --- | --- |
| Primary labor market | Head of state bodies or party, enterprises and institutions, professional and technical personnel, civil servants, clerks and related personnel, businessperson. |
| Secondary labor market | Small retailer, Catering, Housekeeping, Cleaning/janitorial, Security staff, Decoration, Express delivery, Other businesses and services, Primary industry staff, Manufacture, Transportation, Construction, Operator of other manufacturing and transportation equipment, Without fixed occupation, Other |
